# Does Value Lead to Loyalty? Exploring the Important Role of the Tourist–Destination Relationship

**DOI:** 10.3390/bs12050136

**Published:** 2022-05-06

**Authors:** Haihong Wang, Yufan Yang, Wenjun He

**Affiliations:** 1 Department of Tourism Management, School of Business, Liaoning University, Shenyang 110036, China; wanghaihong0209@163.com; 2 School of Business Administration, Faculty of Business Administration, Southwestern University of Finance and Economics, Chengdu 611130, China; yufan.yang@foxmail.com; 3 Department of Marketing and Tourism Management, School of Economics and Management, Wuhan University, Wuhan 430072, China

**Keywords:** perceived value, satisfaction, destination trust, place attachment, loyalty

## Abstract

The perceived value of a tourist’s trip, representing a trade-off between costs and benefits of travelling to a destination, can exert a significant influence on tourists’ loyalty, which is a main concern for managers of tourist destinations. However, the mechanism between the destination value and tourist loyalty remains unexplored, especially in the new context of relationship marketing. To advance the understanding of the mechanism, we introduced 3 variables of “tourist–destination relationship”, namely place attachment (PA), destination trust (DT) and tourist satisfaction (TS), combined with perceived value (PV) and tourist loyalty (TL), and therefore constructed the conceptual model. Taking the Hangzhou City of China as an example, the conceptual model was fitted and tested using the structural equation model (SEM). The results show that: (1) PV can directly and positively affect TS and DT respectively, while there was no significant effect of PV on PA; (2) TS and DT can directly and positively influence TL, but the effect of PA on TL has not been supported empirically; (3) PV has a significant positive effect on TL; and (4) there are internal relationship among the 3 variables measuring the tourist–destination relationship, in other words, TS can significantly affects PA, DT respectively, and DT has a significant positive effect on PA. The findings of this study provide empirical references for understanding the important role of the tourist–destination relationship.

## 1. Introduction

With the continuous development of destinations, tourists have more choices and have become more demanding in the selection of destination, which makes it necessary for destination managers to adopt different marketing strategies to convey attractive messages that will motivate tourists to visit and revisit them [1]. Transactional and relational perspectives have always been used as two different marketing perspectives by destination managers. While the former emphasizes the consumer’s perspective of quality and price [2], namely perceived value, the latter focuses on cultivating a strong connection between the customer and the brand [3]. Many researchers have emphasized the importance of perceived value [4] and relationship quality [5] to tourist loyalty respectively, however, the question of whether there exists an internal relationship between perceived value and relationship quality remains largely unexplored.

On the other hand, there is a growing interest in destination loyalty studies as loyal tourists tend to spend more money (Shoemaker & Lewis, 1999) and provide positive word of mouth [6], which is a key concern for destination managers. Previous studies mainly focus on concepts, antecedents and indicators [7]; for example, Asli et al. modeled an integrated place-oriented and people-oriented concept to explore destination loyalty [8]. Li, Lv and Scott believed that sensory impressions and destination image are two important antecedents of destination loyalty [9]. Cossio-Silva and his colleagues adapted Best’s customer loyalty index to destination loyalty [10]. However, there exists limited research attention on the impact of both tourist–destination relationship [5] and perceived value.

Based on previous research, the present study acknowledges the theoretical and practical gap and aims to fill the gap by introducing elements of perceived value (PV), 3 variables of the tourist–destination relationship (namely tourist satisfaction, destination trust and place attachment, according to Chen and Phou) [5] and tourist loyalty (TL), and thus develop a more comprehensive theoretical model for understanding the formation of tourist loyalty, and address the issue of guiding transactional and relational marketing activities. This study has 2 main aims. First, it investigates the effect of perceived value on tourist loyalty and the tourist–destination relationship. Second, it examines the internal relationship among the 3 variables of the tourist–destination relationship, and their impact on the tourist loyalty.

## 2. Literature Review and Hypothesis Development

### 2.1. Tourist Loyalty and Perceived Value

Tourist loyalty can be divided into a behavioral level and attitudinal level. The former refers to tourists’ participation in activities and measures their repeated and consistent behavior. The latter focuses on describing tourists’ emotional preferences and favoritism towards a destination [11]. A high degree of visitor loyalty dramatically enhances the stability of a destination’s clientele. It provides an effective marketing channel for the destination, that is, publicity through word of mouth, which can significantly increase the destination’s economic revenue [12]. Due to the importance of tourist loyalty, tourist loyalty studies have emerged since the 1980s, and loyalty remains a vital focus for the academic community until today [13].

The academic community has increasingly focused on the importance of perceived value. The concept of perceived value was developed in the 1970s and early 1980s in the retail industry. In the mid-to-late 1990s, the perceived value concept was introduced into tourism research, with Murphy et al. emphasizing that the perceived value of a destination represents the weighting of a tourist’s travel time (or investment of money) against the experience gained from the visit [14]. Tourists estimate the value of a destination based on the difference between the perceived benefits and the costs paid for the tourism experience, including the acquisition value and the transaction value [15]. Exploring tourists’ perceived value is therefore crucial for the management of tourism destinations [16]. It helps destination managers better grasp tourists’ needs and gain a deeper understanding of the value and meaning of the products and services offered by the destination. However, whether and how perceived value leads to tourist loyalty has not been adequately explored.

### 2.2. Tourist–Destination Relationship

“Relationship” is a social psychological concept derived from the structural theory of interpersonal relationships [17], and its essence is interdependence over time [18]. According to Blackston, brand relationship is a stable, intimate, and continuous relationship formed during the interaction between a customer and a brand [19]. Muniz et al. further extended the scope of the concept, arguing that brand relationships should include not only customer–brand relationships but also inter-customer relationships and inter-brand relationships, thus proposing a broad brand relationship theory [20]. Since the 1990s, progressively more discerning tourists and increasingly fierce competition have forced marketers and managers to think about how to distinguish their destinations from competitors, deliver positive messages to tourists, and encourage them to repeat their visits. 

Destination branding is undoubtedly a quality solution. Some scholars argue that a successful destination brand needs to meet tourists’ emotional and functional needs, thus creating a positive and active relationship between tourists and the destination [21]. DeBenedetti et al. also suggested that the relationship between tourists and destinations can be inspired by the relationship between customers and brands [22], and Li conducted a study on the tourist–destination relationship from the perspective of destination marketing [23]. Chen and Phou conducted an empirical study focusing on the tourist–destination relationship, which consists of three dimensions (satisfaction, trust, and attachment), and their conclusions confirmed the hypothesis that the better the relationship between tourists and destinations, the higher their loyalty [5]. Kumar et al. measured the strength of destination relationships using these three variables (satisfaction, trust, and attachment) in their study on destination personality, self-consistency, and tourist loyalty [24].

### 2.3. Hypothesis Development

A large body of research in tourism confirms the link between tourists’ perceived values, attitudes, and behaviors [25]. For example, Han and Hyun demonstrated a positive relationship between perceived quality, tourist satisfaction, tourist trust, and loyalty [26]. Wu et al. showed that environmental quality, relationship quality, and interaction quality positively contribute to medical tourists’ trust, satisfaction, and behavioral intentions [27]. That is, the higher the perceived benefits and the lower the perceived costs, the more likely a tourist is to develop satisfaction, trust, and attachment to the destination and form a close relationship with it. Therefore, the study concluded that perceived value positively influences destination relationships. Among the three dimensions of the tourist–destination relationship, tourists’ satisfaction can be strongly impacted by the value they perceive [28], and this relationship has been confirmed in studies of destination marketing. For example, Chen and Chen focused on tourist behavior in heritage tourism destinations and demonstrated that tourists’ perceived value of the destination plays a significant role in their satisfaction levels [29]. Pandža et al. similarly found a significant positive effect of perceived value on satisfaction [30]. 

When customers perceive a brand as having a high value, they are more willing to establish a long-term connection with the brand and have confidence in its future development [31]. When the perceived value increases, this emotional attachment also increases, which is another dimension variable of the tourist–destination relationship. While perceived value has been studied intensively in tourism studies, these have focused on how tourism companies can make customers perceive higher tourism benefits, thus increasing tourists’ satisfaction and loyalty. The present study argues that just as perceived value is one of the value bases of destination trust, it also plays a vital role in place attachment. If tourists perceive the costs of tourism to be higher than its benefits, emotional place identity, place dependence, and attachment will cease to exist. 

Finally, the link between perceived value, attitude, and behavior has been emphasized by many scholars in recent years, Doney et al., for example, explored the nature of trust in buyer–seller relationships and argued that customer perceived value plays a value-based role in the trust formation process [32]. Additionally, empirical studies have verified the hypothesis that perceived value has a significant positive effect on trust in destinations [33,34]. Al-Ansi, for example, pointed out that tourists’ perceived value of halal-friendly destinations is essential for their trust. If tourists feel that the benefits of tourism are more significant than the costs, they will trust the products and services more and thus enjoy a good interaction with the destination [25]. Based on the arguments above, Hypotheses 1a–c for this study are presented:

**Hypothesis** **1** **(H1a).** *Perceived value has a positive effect on tourist satisfaction.*

**Hypothesis** **1** **(H1b).** *Perceived value has a positive effect on place attachment.*

**Hypothesis** **1** **(H1c).** *Perceived value has a positive effect on destination trust.*

Among the internal dimensions of the tourist–destination relationship, satisfaction is always associated with positive word of mouth and a higher level of trust [35]. While some scholars argue that customer trust leads to satisfaction, others contend that the level of satisfaction positively contributes to the level of trust [36,37]. Scholars who hold this view believe that the more satisfied customers are with a brand, the more they will trust it [38]. Conversely, a customer who is dissatisfied with products and services may question the competence of the provider of the products and services [39]. A study by Lee et al. found that customer satisfaction is likely to trigger positive feelings towards the manufacturer, increasing trustworthiness [40]. Lai similarly argued that the higher the level of satisfaction of tourists, the higher the level of trust in the destination [35]. 

Research in marketing has demonstrated that if consumers are satisfied with a brand, they may develop an emotional attachment to it [41]. This view is supported by Halpenny, who argued that tourists’ satisfaction with the social, natural, and other park environments promotes their sense of place attachment [42]. Zenker and Rütter stated that place satisfaction is the most significant predictor of place attachment [43]. In their review of the place attachment literature, Chen et al. concluded that place attachment is an outcome of an individual’s evaluation of a destination or attitude toward the destination, and again demonstrated a link between satisfaction and place attachment [44]. Ramkissoon et al. argued that tourists’ satisfaction has a significant indirect effect on their sense of place attachment by encouraging environmentally friendly behavioral intentions [45]. Jia et al. pointed out that tourist satisfaction has a positive relationship with tourists’ identification with a place and their sense of attachment [46]. 

In marketing research, brand trust has been shown to be a vital prerequisite for evoking customers’ emotional attachment to a brand [18], and in tourism research, the empirical findings of Chen and Phou showed that the level of tourists’ trust in a destination has a positive correlation with their level of attachment to that destination [5]. Furthermore, in 2016 Kumar et al. similarly confirmed the role of destination trust on place attachment [24]. Therefore, this study argues that for tourist destinations (especially highly reputable destinations), the more tourists trust the quality of the products and services they provide, the more likely they are to form place attachment. Based on the arguments above, Hypotheses 2a–c for this study are presented:

**Hypothesis** **2** **(H2a).** *Tourist satisfaction has a positive effect on destination trust.*

**Hypothesis** **2** **(H2b).** *Tourist satisfaction has a positive effect on place attachment.*

**Hypothesis** **2** **(H2c).** *Destination trust has a positive effect on place attachment.*

A good relationship with the destination is an essential prerequisite for tourists to remain loyal. When tourists are no longer satisfied, trusting, and attached to the destination, their loyalty level will wane significantly. Moreover, they may evaluate the destination negatively, stop visiting the destination, and discourage others from doing so. As confirmed by Chen and Phou [5], there is a significant positive effect of the tourist–destination relationship on loyalty. Therefore, this study suggests that the destination relationship positively affects tourist loyalty. Many studies have confirmed that tourist satisfaction is an important variable leading to future behavior and intention of tourists. Wang et al. explored the behavior of tourists revisiting harbor attractions in Taiwan. They showed that tourists satisfied with their travel experience were more willing to revisit the same destination in the future [47]. The findings of Chen and Phou also confirm the hypothesis of a positive relationship between tourist satisfaction and loyalty [5], and suggest that a tourist who is satisfied with a destination is more likely to revisit it and recommend it to others, leading to attitudinal loyalty as well as behavioral loyalty [48]. 

A sense of attachment is an important prerequisite for individuals to generate behavioral intentions and an important and valid predictor of tourist loyalty [49]. Tourists who are emotionally willing to connect with a destination are more willing to devote more time, effort, and money to maintaining that relationship and are more likely to develop attitudinal and behavioral loyalty [50]. Many researchers have noted this logical relationship and attempted to develop a research framework of place attachment and to measure its relationship with behavioral intentions. In 2013, Ramkissoon et al. explored the pro-environmental behavior of park visitors, and their results demonstrated that visitors’ sense of place attachment (and social connection to the destination) is an important driver of pro-environmental behavior [49]. Yüksel et al. similarly demonstrated this relationship in their study [51]. Therefore, the relationship between place attachment and tourist loyalty has been clearly established.

Trust is an essential relationship marketing tool to increase loyalty [52], so the study of customer loyalty has become more common. The results of empirical studies by Chaudhuri et al. reaffirmed the importance of brand trust for loyalty and suggested that brand trust makes customers believe that establishing an exchange relationship with a brand leads to high value [53]. In the tourism literature, Loureiro and González also provided evidence that tourist trust positively affects loyalty in rural accommodation [37]. Han and Hyun’s study on medical tourism showed that tourists’ trust in clinics and their staff significantly affects their willingness to revisit [26]. Moreover, the willingness to revisit is included in loyalty, which suggests that a higher level of tourist trust promotes a higher level of attitudinal loyalty. In addition, numerous scholars approaching the issue from different perspectives have argued that tourist trust in a destination has a positive effect on tourist loyalty [25], and that tourists are more likely to form attitudinal and behavioral loyalty. Based on the arguments above, Hypotheses 3a–c for this study are presented:

**Hypothesis** **3** **(H3a).** *Tourist satisfaction has a positive effect on tourist loyalty.*

**Hypothesis** **3** **(H3b).** *Place attachment has a positive effect on tourist loyalty.*

**Hypothesis** **3** **(H3c).** *Destination trust has a positive effect on tourist loyalty.*

Finally, tourism research has widely demonstrated the effect of perceived value on tourist loyalty. Chen et al. noted that perceived value has a significant effect on satisfaction and directly influences tourists’ behavioral intentions [29]. Peña et al. also noted that perceived value positively correlates with loyalty [54]. Therefore, it can be argued that the higher the perceived value and the lower the cost of tourism, the more likely tourists are to view the destination as high in quality and worthy of being visited again. Moreover, they are more likely to think of the products and services of the destination when making decisions about their next holiday, resulting in attitudes and behaviors that reflect tourist loyalty, such as recommendations and repeat visits.

**Hypothesis** **4** **(H4).** *Perceived value has a positive effect on tourist loyalty.*

**Figure 1:**
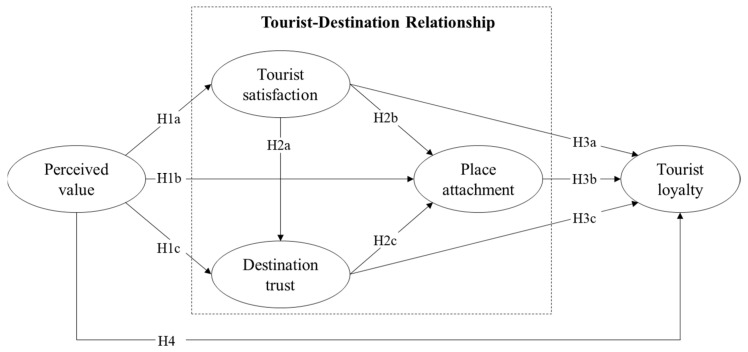
Conceptual model and hypotheses.

All the hypotheses are presented in the conceptual model (Figure 1).

## 3. Methodology

### 3.1. Measures

The questionnaire used for this study consists of two parts. The first part contains demographic characteristics (gender, age, education status and monthly income). The second part involves the measurement of 5 variables: the perceived value, tourist–destination relationship (including tourist satisfaction, destination trust and place attachment) and tourist loyalty. In order to minimize the data overbias in the process of structural equation modeling, we adapted a seven-point Likert scale in the questionnaire, corresponding to measures 1–7 (1 = strongly disagree; 7 = strongly agree).

Measurement items for perceived value and tourist loyalty were adapted from the scale used by Al-Ansi and Han, who analyzed the perceived value and destination loyalty of travel to Korea [25], which can be adapted to the large-scale destination. These 2 measurement scales consist of 4 items respectively (e.g., “Traveling to this destination is worth the price” “I am willing to revisit this destination in the near future”), and the scales yielded Cronbach’s alpha values of 0.90 (perceived value) and 0.91 (tourist loyalty), suggesting good measurement reliability. 

According to Chen and Phou [5], the measurement scales for tourist–destination relationship can be divided into 3 parts: the tourist satisfaction, destination trust and place attachment. The measurement scale for tourist satisfaction was adapted from the 3-item scale developed in Yüksel and Yüksel’s research (e.g., “Overall, I am satisfied with the decision to have my holiday in this destination”), and the Cronbach’s alpha for this scale was 0.94. Similarly, we adapted four items from the 3-item place dependence scale and 3-item affective attachment scale in Yüksel and Yüksel’s study, two items from the scales were dropped because of awkwardness and ambiguity reported by pilot participants, and the Cronbach’s alpha for this scale was 0.82. Finally, the five items measuring destination trust (e.g., “This destination is a destination that never disappoints me”) were adapted from the eight-item scale developed in Chen and Phou [5]. We dropped the other three items because participants in the pilot study perceived them as awkward and confusing. With a Cronbach’s alpha value of 0.92, this scale showed good measurement reliability as well. A detailed description of the items is presented in Table 1.

### 3.2. Study Site and Data Collection

Hangzhou, the capital of Zhejiang Province of China, has been called “Heaven on Earth” since ancient times due to its rich and high-quality tourism resources and pleasant climate conditions. There are 103 A-class scenic spots in the city, including three 5A-class scenic spots like West Lake. It is a destination with strong tourism competitiveness, and destination managers and researchers can learn a lot about tourism development by studying Hangzhou’s example. Therefore, this study takes Hangzhou as a case study site to study tourist perceived value, the tourist–destination relationship, and tourist loyalty. As research subjects we selected tourists who were non-residents of Hangzhou and visited Hangzhou for sightseeing and vacation during 2018–2019, taking the impact of COVID-19 into account. Furthermore, we commissioned the Credamo platform, which is an online professional platform that provides data sample collection and allows a 30% questionnaire re-delivery rate, to conduct online research from 6 to 10 April 2020.

To ensure the quality of the questionnaire answers collected, we conducted a presurvey with 3 graduate students majoring in tourism management before the formal questionnaire, asking them to examine and assess the content and provided feedback on and suggestions for improvement, and we timed the average time spent (97 s) as an exclusion criterion and revised the questions according to the feedback. The final form of the questionnaire was used to collect data, and 250 questionnaires were distributed. After eliminating invalid questionnaires (not completed, not objective), 213 valid questionnaires were obtained, while 37 questionnaires were marked as invalid, and therefore needing re-collection. After the second-round data collecting, we obtained 248 valid data samples in all. Finally, 28 questionnaires that taken in less than 97 s were excluded because it is believed that non-professional subjects need to spend more time understanding the questions than the professional subjects. Therefore, 220 valid data samples were obtained, with an efficiency rate of 88.0%. This sample size was deemed to be sufficient considering the low model complexity as well as the reliability and validity of adopted measurements [55].

## 4. Data Analysis

### 4.1. Preliminary Analysis

Of the 220 respondents, 24.55 percent were male, and 75.45 percent were female. The main age range was between 18 and 35 years. A more detailed demographic profile of the participants is presented in Table 2.

Assessments of potential multivariate normality issues were performed by evaluating skewness and kurtosis of each item distribution. The skewness values ranged from 0.12 to 1.49; the kurtosis values ranged from 0.89 to 3.40. As none of the absolute skewness values were equal to or greater than 2, and none of the absolute kurtosis values were equal to or greater than 7, it was deemed that the distribution was not significantly kurtotic [56]. Nevertheless, as the later structural equation modeling technique is based on the analysis of covariance, which is sensitive to kurtotic distributions, maximum likelihood estimation with bootstrapping (5000 bootstrap samples with bias-corrected confidence intervals of 95%) was used in subsequent analyses, given that these techniques are considered more robust to moderate non-normality data issues [55].

### 4.2. Structural Equation Modeling (SEM) Analysis

#### 4.2.1. Measurement Model Assessment

The measurement model was assessed through a two-step SEM analysis following recommendations in Anderson and Gerbing via SPSS AMOS 24 with maximum likelihood estimation and bootstrapping [57]. The first step focused on measurement validity through confirmatory factor analysis (CFA), and the second step focused on assessing structural relationships simultaneously for hypothesis testing. The measurement model yielded a good fit with χ^2^ = 493.07 (*p* < 0.001, df = 160); χ^2^/df = 3.08; comparative fit index (CFI) = 0.92; Tucker–Lewis index (TLI) = 0.90; and standardized root mean square residual (SRMR) = 0.05. In addition, the root mean square error of approximation (RMSEA) was 0.09. As shown in Table 1, all standardized factor loadings for each item were statistically significant (*p* < 0.001) and in the 0.62 to 0.94 range, reflecting good reliability [55].

Convergent validity was assessed by evaluating the average variance extracted (AVE) values for all constructs (Table 3). All AVE values were above 0.5, suggesting that the majority of the variance in each construct was explained by the adopted measurement items. In addition, the composite reliability (CR) values of all constructs were above 0.8, showing good convergent validity [55]. With respect to discriminant validity, the square root of each AVE value (as shown in the correlation matrix in bold and italics)—except for place attachment—was larger than the corresponding cross-correlations, demonstrating good discriminant validity [58]. With an adequate measurement model fit achieved, the following structural relationship analyses were conducted.

#### 4.2.2. Structural Model Assessment

**Figure 2:**
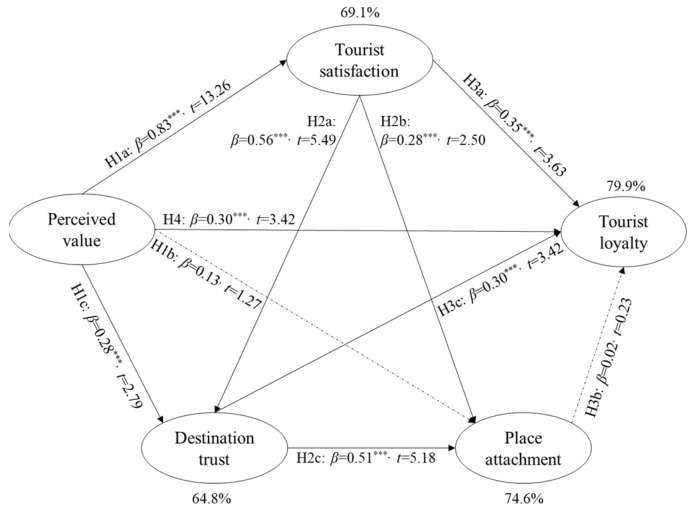
Results of the hypotheses test. Note. *** *p* < 0.001.

The hypothesized relationships were tested through SPSS AMOS 24 with maximum likelihood estimation. The structural model also achieved a good fit with χ^2^ = 493.07 (*p* < 0.001, df = 160); χ^2^/df = 3.08; CFI = 0.92; TLI = 0.90; RMSEA = 0.09; and SRMR = 0.05. All hypotheses except Hypothesis 1b (perceived value → place attachment) and Hypothesis 3b (place attachment → tourist loyalty) were supported, as shown in Figure 2 and Table 4. The results suggest that perceived value contributed significantly to tourist satisfaction as well as destination trust (Hypothesis 1a: β = 0.83, *t* = 13.26 ***; Hypothesis 1c: β = 0.28, *t* = 2.79 **). Tourist satisfaction significantly contributed to place attachment (Hypothesis 2b: β = 0.28, *t* = 2.50 **) and destination trust (Hypothesis 2a: β = 0.56, *t* = 5.49 ***), and trust can significantly affect place attachment (Hypothesis 2c: β = 0.51, *t* = 5.18 ***) and tourist loyalty (Hypothesis 3c: β = 0.30, *t* = 3.20 ***). The result also proves that tourist loyalty can be affected by tourist satisfaction (Hypothesis 3a: β = 0.35, *t* = 3.63 ***) and perceived value (Hypothesis 4: β = 0.30, *t* = 3.42 ***). Overall, this model explained 69.1% of variance in tourist satisfaction, 64.8% of variance in destination trust, 74.6% of variance in place attachment, and 79.9% of variance in tourist loyalty. 

## 5. Discussion and Implications

### 5.1. Discussion

The existing tourism literature emphasizes the important role of perceived value as well as the tourist–destination relationship for better understanding tourist behavior [5]. Nevertheless, these studies are limited to focus on how the perceived value or the tourist–destination relationship affects tourist loyalty. Therefore, regarding the need for research on this issue, this study is aimed to explored both the role of perceived value and tourist–destination relationship in the process of cultivating loyal tourists, which can provide an association among the perceived value, tourist–destination relationship, and tourist loyalty. Overall, this study identified 4 major findings, explained in details below.

First, perceived value can directly and positively influence tourist loyalty, which validates Hypothesis 4 of this study. This is perhaps because the perceived value is a comprehensive trade-off between gains and costs, and tourists take the initiative to choose high-quality destination in their future decisions, which reflects both attitudinal and behavioral loyalty.

This study also identified the impact of perceived value on the tourist–destination relationship. Consistent with the results of previous studies, the empirical results show that a higher perceived value leads to higher satisfaction [5], which validates Hypothesis 1a of this study. This reflects the fact that tourists usually compare what they gain from the destination with what they paid, and this comparison can have a crucial impact on tourists’ satisfaction. Thus, destinations can better satisfy tourists when they offer high value to meet tourists’ needs and expectations. 

Furthermore, this study also proves that perceived value positively affects the level of trust in the destination (H1c). As discussed in the previous research, perceived value is the value basis of trust [32], in other words, if tourists regard a destination as worthwhile, it means that they believe they can have a high-quality tourist experience in this destination, which motivates tourists’ trust. This also complements Baloglu’s findings, which showed that cognitive factors (e.g., perceived value) have a significant impact on affective factors (e.g., destination trust) [59]. 

However, the perceived value does not have a positive impact on the place attachment (H1b), with a small and insignificant coefficient of 0.13, which implies that it is challenging to create place attachment even when tourists perceive the destinations as having high value. This may be due to the difference between the cognitive judgement and the emotional attachment, with perceived value focusing on tangible products and attachment focusing on the intangible experiences. Furthermore, tourists’ memory of a journey is short-term, quickly fading after returning to their daily lives. Thus, it is difficult for tourists to develop a long-term place attachment even with a high-value destination.

This study further explored the internal effects among the 3 variables of tourist–destination relationship (namely tourist satisfaction, destination trust, and place attachment), and the empirical results validate H2a–c. Firstly, the finding that tourist satisfaction can positively affect destination trust (H2a) is consistent with Lai’s [35] finding that higher satisfaction leads to higher destination trust. This is because tourists’ satisfaction level reflects their judgement on the tourism products and services of the destination, and unsatisfied tourists tend to question whether they can have a nice experience in this destination, thus regarding the destination’s products and services as unreliable and untrustworthy. 

The results also show that tourist satisfaction has a positive effect on the place attachment (H2b). These results are in line with the findings of the previous studies, which also found that satisfaction is an essential factor influencing place attachment [41,46]. This may be because tourist satisfaction is a subjective judgment when the total gain by tourists is greater than the expected gain. When tourists are satisfied, they are more likely to identify with local customs and the history and culture of the destination, and more willing to establish an emotional connection with the destination and thus generate attachment. 

This study also finds that destination trust has a positive effect on the place attachment (H2c). This finding is consistent with the studies of Esch et al. [18], Chen et al. [5], and Kumar et al. [24], which may be because trust itself is an intimate dependency relationship, and when tourists trust the products and services of a certain destination, they are more likely to want to establish a long-term connection with the destination. 

Finally, the empirical result shows that tourist satisfaction (H3a) as well as destination trust (H3c) has a significant and positive effect on tourist loyalty, while the place attachment doesn’t have a significant impact on the loyalty (H3b). The results that satisfaction and trust can affect tourist loyalty are in line with the findings of the previous studies, which also found that tourist satisfaction and trust are essential antecedent variables of tourist loyalty [52]. 

However, we also find that tourists’ place attachment does not significantly and positively affect tourists’ loyalty to the destination, this is perhaps because the destination chosen is in eastern China, where a wide range of tourism resources are concentrated. Although tourists to Hangzhou have a certain level of local attachment, they are less likely to revisit because tourists always seek diversity in their tourism experiences, thus reducing the overall level of loyalty.

### 5.2. Implications

With an increasing number of rational tourists faced with an ever-expanding number of destination choices, a given tourism destination management organization can dominate most thoroughly by providing tourists with increased value as compared to the competition. Thus, destination managers should continue to improve the quality of products and services to increase the overall value perceived by tourists, which is crucial for destination branding. For example, managers can expedite the construction of infrastructure to ensure the destination’s basic functional value. In addition, they should improve the quality of products and services to enhance tourists’ perception of quality by improving their experience and feelings. Finally, managers should try to reduce the costs of tourists before and during tourists’ visit, not only the monetary cost but also the amount of time required. For example, additional tourist lines can be set up to ensure the accessibility of the destination, thus reducing the cost and duration of transportation, which can improve tourists’ perceived value.

However, some destination management organizations only focus on the quality of services and products but ignore the importance role of the tourist–destination relationship, which plays a crucial role in the formation of tourist loyalty and in the process of destination branding. The results of our empirical analysis illustrate that perceived value can directly affect tourist loyalty. However, the impact is not as significant as trust and satisfaction, which are two measurement variables of the tourist–destination relationship. This reflects that the value is the basis of a good relationship, and the good relationship is an extension of value in turn. Therefore, it is recommended that managers of tourist destinations, under the premise of ensuring that tourists have a perception of high quality about the destination, shift their branding strategy to the tourist–destination relationship.

Furthermore, it is essential to pay attention to the emotional connection that tourists form with destinations and ensure that this connection endures. As Pike et al. have argued, for destinations management organizations, similar and substitutable tourism products are not enough to attract tourists, so it is necessary to focus on key variables of the destination relationship, such as satisfaction, destination trust, and place attachment [60]. This lasting and positive emotional bond between tourists and destinations can help tourists better differentiate a destination from its competitors, thus increasing its destination brand competitiveness. This requires destination managers to create a desirable perception of the destination brand through public relations management, enhance the reliability of the destination, and motivate tourists’ behavioral intentions. Moreover, destination managers should take the initiative to establish contact with tourists, use new media platforms such as Weibo and Douyin to increase the frequency and depth of interaction with tourists. Finally, tourism managers can build feedback channels to respond quickly and reasonably to the problems tourists encounter while at the destination to improve their satisfaction and trust.

## 6. Limitations and Future Research

The findings of this study will help destination managers understand the effects of perceived value and the tourist–destination relationship on tourist loyalty, while several limitations provide potential avenues for future research. First, this study examined tourists from Hangzhou, thus limiting the tourist destinations studied to geographically proximal provinces such as Jiangsu, Shanghai, and Jiangxi. Whether the findings of this study apply to different tourist destinations needs to be verified. Especially in the context of rural revitalization, it is of great value to explore the formation mechanism of tourist loyalty by taking the tourist–destination relationship to improve rural entrepreneurial performance [61]. Second, as the personality of travellers can largely predict the travel intention and revisit intention [62], it is interesting and valuable to explore how the relationship between the perceived value and tourist loyalty is affected by personality variables of the travellers. Furthermore, although we have taken the huge impact of COVID-19 into account, the empirical results were inevitably affected by the pandemic, and further research can also examine the impact of COVID-19 on tourists’ perceived value and the tourist–destination relationship. And finally, this study used a survey method to collect data and a structural equation modeling technique to analyse the data, and therefore, there may be a common method variance problem. Future research may use a technique such as between-subject experiment to minimize the problem.

## Figures and Tables

**Table 1:** Measurement and standardized loading information.

Item	Description	Standardized Loading	*t*-Value
Perceived value (coded as PV)			
PV1	Traveling to Hangzhou is worth the price.	0.85	-
PV2	Compared to other destinations, traveling to Hangzhou is a good deal.	0.78	13.62
PV3	Traveling to Hangzhou offers good value for money.	0.86	16.00
PV4	Traveling to Hangzhou meets my travel needs.	0.88	16.60
Tourist satisfaction (coded as TS)			
TS1	I am happy about my decision to stay in Hangzhou	0.90	-
TS2	I believe I did the right thing when I chose to make my holiday in Hangzhou	0.94	22.81
TS3	Overall, I am satisfied with the decision to make my holiday in Hangzhou	0.94	22.87
Place attachment (coded as PA)			
PA1	Hangzhou means a lot to me.	0.72	-
PA2	I am very attached to Hangzhou.	0.78	10.80
PA3	For the activities that I enjoy most, the settings and facilities provided by Hangzhou are the best.	0.62	8.55
PA4	For what I like to do, I could not imagine anything better than the settings and facilities provided by Hangzhou.	0.80	11.08
Destination trust (coded as DT)			
DT1	Hangzhou guarantees tourist satisfaction.	0.85	-
DT2	Hangzhou is a destination that never disappoints me.	0.87	16.88
DT3	Hangzhou would compensate me in some ways for the problems with the trip.	0.80	14.68
DT4	Hangzhou would make any effort to satisfy tourists.	0.83	15.35
DT5	Hangzhou would be honest and sincere in addressing my concerns.	0.83	15.57
Tourist loyalty (coded as TL)			
TL1	I am willing to revisit Hangzhou in the near future.	0.74	-
TL2	I intend to visit Hangzhou again in the near future.	0.80	12.11
TL3	I am willing to recommend other people to visit Hangzhou.	0.92	14.07
TL4	I will say positive things to other people about Hangzhou as a tourist destination.	0.90	13.70

**Table 2:** Profile information of participants (*n* = 220).

Variable	Category	Count	Percentage
Gender	Male	54	24.55
	Female	166	75.45
Age	18–25	96	43.64
	26–35	92	41.82
	36–45	30	13.64
	46–55	2	0.91
	55 and above	0	0.00
Education status	Primary school and below	0	0.00
	Junior School	1	0.45
	High School/Technical secondary school	9	4.09
	Junior college/Higher vocational college	17	7.73
	Undergraduate college	137	62.27
	Postgraduate and above	56	25.45
Monthly income	Under 2000	87	39.55
(RMB)	2001–5000	54	24.55
	5001–8000	46	20.91
	8001–10,000	19	8.64
	10,001 and above	14	6.36

**Table 3:** CR, AVE, and correlation matrix.

	CR	AVE	1	2	3	4	5
1. PV	0.91	0.71	0.84				
2. TS	0.95	0.85	0.83	0.92			
3. PA	0.82	0.54	0.75	0.80	0.73		
4. DT	0.91	0.71	0.74	0.79	0.83	0.84	
5. TL	0.92	0.70	0.82	0.85	0.77	0.81	0.84

**Table 4:** Hypothesis testing results.

Hypothesized Paths		Coefficient	*t*-Value	*p*	Result
H1a:	Perceived value → Tourist satisfaction	0.83	13.26	0.00	Supported
H1b:	Perceived value → Place attachment	0.13	1.27	0.20	Not Supported
H1c:	Perceived value → Destination trust	0.28	2.79	0.01	Supported
H2a:	Tourist satisfaction → Destination trust	0.56	5.49	0.00	Supported
H2b:	Tourist satisfaction → Place attachment	0.28	2.50	0.01	Supported
H2c:	Destination trust → Place attachment	0.51	5.18	0.00	Supported
H3a:	Tourist satisfaction → Tourist loyalty	0.35	3.63	0.00	Supported
H3b:	Place attachment → Tourist loyalty	0.02	0.23	0.82	Not Supported
H3c:	Destination trust → Tourist loyalty	0.30	3.20	0.00	Supported
H4:	Perceived value → Tourist loyalty	0.30	3.42	0.00	Supported

Note. R^2^ values for tourist satisfaction, destination trust, place attachment and tourist loyalty were 69.1%, 64.8%, 74.6% and 79.9% respectively.

## Data Availability

The data analyzed in this paper are proprietary, and therefore cannot be posted online.

## References

[1] Roodurmun J., Juwaheer T.D. (2010). Influence of trust on destination loyalty—An empirical analysis-the discussion of the research approach. Int. Res. Symp. Serv. Manag..

[2] Patterson P.G., Spreng R.A. (1997). Modelling the relationship between perceived value, satisfaction and repurchase intentions in a business-to-business, services context: An empirical examination. Int. J. Serv. Ind. Manag..

[3] Sheth J. (2017). Revitalizing relationship marketing. J. Serv. Mark..

[4] Jin N., Lee H., Lee S. (2013). Event Quality, Perceived Value, Destination Image, and Behavioral Intention of Sports Events: The Case of the IAAF World Championship, Daegu, 2011. Asia Pac. J. Tour. Res..

[5] Chen C.F., Phou S. (2013). A closer look at destination: Image, personality, relationship, and loyalty. Tour. Manag..

[6] Oppermann M. (2000). Tourism destination loyalty. J. Travel Res..

[7] Qu Y., Li T. (2010). A review about the loyalty of tourists in tourism destination overseas in the past decade. Tour. Trib..

[8] Tasci A.D., Uslu A., Stylidis D., Woosnam K.M. (2021). Place-Oriented or People-Oriented Concepts for Destination Loyalty: Destination Image and Place Attachment versus Perceived Distances and Emotional Solidarity. J. Travel Res..

[9] Li C., Lv X., Scott M. (2021). Understanding the dynamics of destination loyalty: A longitudinal investigation into the drivers of revisit intentions. Curr. Issues Tour..

[10] Cossío-Silva F.J., Revilla-Camacho M.Á., Vega-Vázquez M. (2019). The tourist loyalty index: A new indicator for measuring tourist destination loyalty?. J. Innov. Knowl..

[11] Backman S.J., Crompton J.L. (1991). The usefulness of selected variables for predicting activity loyalty. Leis. Sci..

[12] Shoemaker S., Lewis R.C. (1999). Customer loyalty: The future of hospitality marketing. Int. J. Hosp. Manag..

[13] Cole S.T., Scott D. (2004). Examining the mediating role of experience quality in a model of tourist experiences. J. Travel Tour. Mark..

[14] Murphy P.E., Pritchard M., Smith B. (2000). The destination product and its impact on traveler perceptions. Tour. Manag..

[15] Zeithaml V.A. (1988). Consumer perceptions of price, quality, and value: A means-end model and synthesis of evidence. J. Mark..

[16] Agapito D., Valle P., Mendes J. (2014). The sensory dimension of tourist experiences: Capturing meaningful sensory-informed themes in Southwest Portugal. Tour. Manag..

[17] Wu Y. (2013). Study on the Impact of Brand Equity and Brand Relationship on Customer Repurchase Behavior in Economic Hotel. Master’s Thesis.

[18] Esch F.R., Langner T., Schmitt B.H., Geus P. (2006). Are brands forever? How brand knowledge and relationships affect current and future purchases. J. Prod. Brand Manag..

[19] Blackston M. (1992). Observations: Building brand equity by managing the brand’s relationships. J. Advert. Res..

[20] Muniz A.M., O’Guinn T.C. (2001). Brand community. J. Consum. Res..

[21] Ekinci Y. (2003). From destination image to destination branding: An emerging area of research. e-Rev. Tour. Res..

[22] DeBenedetti A., Oppewal H., Zeynep A. (2014). Place attachment in commercial settings: A gift economy perspective. Adv. Consum. Res..

[23] Li Y., Guan X. (2015). From “people-brand” Relationship to “people-destination” relationship: The effects of self-destination connection. Tour. Trib..

[24] Kumar V. (2016). Examining the role of destination personality and self-congruity in predicting tourist behavior. Tour. Manag. Perspect..

[25] Al-Ansi A., Han H. (2019). Role of halal-friendly destination performances, value, satisfaction, and trust in generating destination image and loyalty. J. Destin. Mark. Manag..

[26] Han H., Hyun S.S. (2015). Customer retention in the medical tourism industry: Impact of quality, satisfaction, trust, and price reasonableness. Tour. Manag..

[27] Wu H.C., Li T., Li M.Y. (2016). A study of behavioral intentions, patient satisfaction, perceived value, patient trust and experiential quality for medical tourists. J. Qual. Assur. Hosp. Tour..

[28] Li H., Zhou L., Zhen Y. (2018). The influence of tourists’ motivation and perceived value on their satisfaction and willingness to act in “foreign caravanserai”. J. Zhejiang Univ. (Sci. Ed.).

[29] Chen C.F., Chen F.S. (2010). Experience quality, perceived value, satisfaction, and behavioral intentions for heritage tourists. Tour. Manag..

[30] Pandža Bajs I. (2013). Tourist perceived value, relationship to satisfaction, and behavioral intentions. J. Travel Res..

[31] Fan L., Li X. (2018). Brand emotional attachment and brand trust: Moderating effect based on brand familiarity. Ind. Eng. Manag..

[32] Doney P.M., Cannon J.P. (1997). An examination of the nature of trust in buyer-seller relationship. J. Mark..

[33] Guo A., Huang F., Li W. (2013). Empirical research on the key driving factors of revisiting intention: A comparison of perceived value, perceived attraction, tourist satisfaction and tourist trust. J. Jiangxi Univ. Financ. Econ..

[34] Liu W., Lin D. (2018). Research on trust-based word-of-mouth recommendation mechanism of tourism destinations. Tour. Trib..

[35] Lai Q. (2016). Research on the Influence of Tourist Destination Brand Experience on Tourist Loyalty. Master’s Thesis.

[36] Delgado-Ballester E., Luis Munuera-Alemán J. (2001). Brand trust in the context of consumer loyalty. Eur. J. Market..

[37] Loureiro S.M.C., González F.J.M. (2008). The importance of quality, satisfaction, trust, and image in relation to rural tourist loyalty. J. Travel Tour. Mark..

[38] Lee J.S., Back B.J. (2008). Attendee-based brand equity. Tour. Manag..

[39] Ha H.Y., Perks H. (2005). Effects of consumer perceptions of brand experience on the web: Brand familiarity, satisfaction, and brand trust. J. Consum. Behav..

[40] Lee D., Moon J., Kim Y.J., Yi M.Y. (2015). Antecedents, and consequences of mobile phone usability: Linking simplicity and interactivity to satisfaction, trust, and brand loyalty. Inf. Manag..

[41] Thomson M., McInnis D., Park W. (2005). The ties that bind: Measuring the strength of consumers’ emotional attachment to brands. J. Consum. Psychol..

[42] Halpenny E. (2006). Environmental Behavior, Place Attachment and Park Visitation: A Case Study of Visitors to Point Pelee National Park. Ph.D. Thesis.

[43] Zenker S., Rütter N. (2014). Is satisfaction the key? The role of citizen satisfaction, place attachment and place brand attitude on positive citizenship behavior. Cities.

[44] Chen N., Dwyer L., Firth T. (2014). Effect of dimensions of place attachment on residents’ word-of-mouth behavior. Tour. Geogr..

[45] Ramkissoon H., Mavondo F.T. (2015). The satisfaction-place attachment relationship: Potential mediators and moderators. J. Bus. Res..

[46] Jia Y., Lin D. (2016). Tourists’ service perception, place attachment and loyalty: A case study of Xiamen. Geogr. Res..

[47] Wang Y.J., Wu C., Yuan J. (2010). Exploring visitors’ experiences and intention to revisit a heritage destination: The case of Lukang, Taiwan. J. Qual. Assur. Hosp. Tour..

[48] Chen C.F., Tsai D. (2007). How destination image and evaluative factors affect behavioral intentions. Tour. Manag..

[49] Ramkissoon H., Smith L.D.G., Weiler B. (2013). Relationships between place attachment, place satisfaction and pro-environmental behaviour in an Australian national park. J. Sustain. Tour..

[50] Huang C.C. (2017). The impacts of brand experiences on brand loyalty: Mediators of brand love and trust. Manag. Decis..

[51] Yüksel A., Yüksel F., Bilim Y. (2010). Destination attachment: Effects on customer satisfaction and cognitive, affective, and conative loyalty. Tour. Manag..

[52] Berry L.L. (1995). Relationship marketing of services-growing interest, emerging perspective. J. Acad. Mark. Sci..

[53] Chaudhuri A., Holbrook M.B. (2001). The chain effects from brand trust and brand affect to brand performance: The role of brand loyalty. J. Mark..

[54] Peña A.I.P., Jamilena D.M.F., Molina M.Á.R. (2012). The perceived value of the rural tourism stay and its effect on rural tourist behavior. J. Sustain. Tour..

[55] Hair J.F., Black W.C., Babin B.J., Anderson R.E., Tatham R.L.L. (2006). Multivariate Data Analysis.

[56] Byrne B. (2013). Structural Equation Modeling with EQS.

[57] Anderson J.C., Gerbing D.W. (1988). Structural equation modeling in practice. Psychol. Bull..

[58] Fornell C., Larcker D.F. (1981). Structural equation models with unobservable variables and measurement error: Algebra and statistics. J. Mark. Res..

[59] Baloglu S. (1998). An empirical investigation of attitude theory for tourist destinations: A comparison of visitors and nonvisitors. J. Hosp. Tour. Res..

[60] Pike S., Ryan C. (2004). Destination positioning analysis through a comparison of cognitive, affective, and conative perceptions. J. Travel Res..

[61] Wang H., Cao N., Li Z., He Y. (2019). Research on influencing factors of farmers’ entrepreneurial performance in rural tourism industry. J. Liaoning Univ. (Philos. Soc. Sci. Ed.).

[62] Morar C., Tiba A., Basarin B., Vujičić M., Valjarević A., Niemets L., Gessert A., Jovanovic T., Drugas M., Grama V. (2021). Predictors of Changes in Travel Behavior during the COVID-19 Pandemic: The Role of Tourists’ Personalities. Int. J. Environ. Res. Public Health.

